# Breathing Signature as Vitality Score Index Created by Exercises of Qigong: Implications of Artificial Intelligence Tools Used in Traditional Chinese Medicine

**DOI:** 10.3390/jfmk4040071

**Published:** 2019-12-03

**Authors:** Junjie Zhang, Qingning Su, William G. Loudon, Katherine L. Lee, Jane Luo, Brent A. Dethlefs, Shengwen Calvin Li

**Affiliations:** 1 School of Physical Training and Physical Therapy, Shenzhen University, 3688 Nanhai Avenue, Nanshan District, Shenzhen 518060, China; 2 Center of Bioengineering, School of Medicine, Shenzhen University, 3688 Nanhai Avenue, Nanshan District, Shenzhen 518060, China; 3 Neuroscience Institute, Children’s Hospital of Orange County, Gamma Knife Center of Southern California, Department of Neurosurgery, University of California-Irvine School of Medicine, Orange, CA 92612, USA; 4 School of Social Ecology, University of California-Irvine, 5300 Social and Behavioral Sciences Gateway, Irvine, CA 92697-7050, USA; 5 AB Sciex, Inc., Danaher Corporation, 250 South Kraemer Boulevard, Brea, CA 92821-6232, USA; 6 CHOC Children’s Research Institute, Children’s Hospital of Orange County (CHOC), 1201 W. La Veta Ave., Orange, CA 92868-3874, USA; 7 Neuro-Oncology and Stem Cell Research Laboratory (NSCL), CHOC Children’s Research Institute (CCRI), Children’s Hospital of Orange County (CHOC), 1201 W. La Veta Ave., Orange, CA 92868-3874, USA; 8 Department of Neurology, University of California-Irvine (UCI) School of Medicine, 200 S Manchester Ave Ste 206, Orange, CA 92868, USA

**Keywords:** Qigong, breathing signature, vitality score index, AI Medicine, AI deep learning, immune, inflammation, tissue microenvironment, holistic care, telomerase activity, traditional Chinese medicine (TCM)

## Abstract

Rising concerns about the short- and long-term detrimental consequences of administration of conventional pharmacopeia are fueling the search for alternative, complementary, personalized, and comprehensive approaches to human healthcare. Qigong, a form of Traditional Chinese Medicine, represents a viable alternative approach. Here, we started with the practical, philosophical, and psychological background of Ki (in Japanese) or Qi (in Chinese) and their relationship to Qigong theory and clinical application. Noting the drawbacks of the current state of Qigong clinic, herein we propose that to manage the unique aspects of the Eastern ‘non-linearity’ and ‘holistic’ approach, it needs to be integrated with the Western “linearity” “one-direction” approach. This is done through developing the concepts of “Qigong breathing signatures,” which can define our life breathing patterns associated with diseases using machine learning technology. We predict that this can be achieved by establishing an artificial intelligence (AI)-Medicine training camp of databases, which will integrate Qigong-like breathing patterns with different pathologies unique to individuals. Such an integrated connection will allow the AI-Medicine algorithm to identify breathing patterns and guide medical intervention. This unique view of potentially connecting Eastern Medicine and Western Technology can further add a novel insight to our current understanding of both Western and Eastern medicine, thereby establishing a vitality score index (VSI) that can predict the outcomes of lifestyle behaviors and medical conditions.

## 1. Background

Breathing is essential for life, as shown by the 2019 Nobel Prize in Physiology or Medicine awarded jointly to cancer researcher William G. Kaelin Jr. (O_2_ sensing, VHL gene [1]), physician-scientist Sir Peter J. Ratcliffe (HIF-mediated hypoxia signaling, [2]), and geneticist Gregg L. Semenza (hypoxia and cancer, [3]) “for their discoveries of how cells sense and adapt to oxygen availability.” Life is as good as breathing, as organisms sense O_2_ levels crucial not only for a developing fetus but also for wellness of a mature individual, as well as for tumor growth. Lack of breathing (deprivation of O_2_) leads to hypoxia, causing severity such as fetus death, ischemic heart (myocardial infarction [4], and stroke ([5]), as well as brain injury (learning and memory deficits that are related to loss of CA3 pyramidal neurons [6]). Reflecting on history, researchers can track back to “The *Principles of Yellow Emperor’s Internal Medicine*” (in Chinese pinyin: HuangdiNeijing 《黄帝内经》), known as the Bible of Traditional Chinese Medicine (TCM), which was published over 2500 years ago, as a 200,000-word definitive document [7]. This classic reference defines specific pathways of energy flow within the human body, the meridian channels, and their connections. Interestingly, meridian networks are reproduced by identified TCM practitioners but have thus far defied identification by western diagnostic technologies.

Qi, known as a force, an energy flow, a fluid in the body, is a fundamental concept in TCM, Eastern medicine, and Eastern philosophy. The Qi of the human body flows along these meridians that conduct the inter-organ electrical conductance, by which Qigong increases physiological functions significantly (*p* < 0.05). As an example, a significant positive correlation exists between kidney meridian electrical conductance and pericardium meridian electrical conductance, compared with baseline values of the non-practitioners [8]. The human body becomes ill when the normal Qi flow is perturbed. Qigong represents an ancient Chinese therapeutic modality focused upon normalizing and sustaining healthy Qi flow through these meridians.

According to textual research, the word “Qigong” was first conceived in the book *Jingming Religious Record* 《净明宗教录》 written by Xu Xun (许逊), a Taoist scholar during the Jin Dynasty (A.D. 266–420). In general, breathing force, i.e., Qi (oxygen flow along with bloodstream), in the human body is analogous to the potential energy of water on a high mountain, flowing down defined channels traveling through defined routes. Qigong exercises purportedly facilitate the momentum of “Qi,” normalizing energy streaming through meridians, promoting health.

As part of TCM practice, Qigong is a body-mind coordinating exercise that combines body- adjustment, breathing-adjustment, and mind-adjustment. In terms of modern medicine of behavioral therapy, Qigong exercise targets behavioral learning and training that is beneficial to mental and physical health. With practice, Qigong becomes an established conditioned reflex behavior, promoting therapeutic impact.

So, what exactly does Qigong Exercise involve? Qigong consists of two elements: “Qi” (breathing) and “gong” (the functional consequence of Qi practice). The answer is complicated as many schools of Qigong practitioners (hardcore Qigong, Kungfu Qigong, soft Qigong, Tai Chi Qigong, Buddha Qigong) have been documented through 2000 years fundamentally. The practice of Qigong is involved in its breadth of average health, by synchronizing breathing and movement. It is characterized by practiced efforts to integrate body-adjustment, mind-adjustment, breathing-adjustment, and other physical activities for a state of harmony, realizing mind, body, and spiritual equilibrium. For example, Buddha Qigong practitioner can go through the following five levels of sight in accomplishment (the functional consequence of Qi practice): First level: The practitioner can only see things that are in front of them, not what is behind them. The second level is to Open the Heavenly Eye (or Third Eye) which enables the practitioner to see more than what is in front of them, such as seeing through things and seeing far away. Third level is to Open the Wisdom Eye, including “Many Eyes” (they can see the same thing from different perspectives), “Analyzing Eye” (which can not only see something but understand it), “Chasing Eye” (when one can trace something back to whom it belongs), and “Predicting Eye” (they can see the future). The forth level is to Open the Universal Eye (they can move things around without touching them (telekinesis)). The fifth level is to Open the Buddha’s Eye (they can bring healing effects to anyone who approaches them without them even intending to do so. Their possessions have the same healing properties, e.g., their pens, business cards, and clothes can all be used to treat disease) (http://www.qigongchinesehealth.com/5 levels of sight, as accessed on 11 October 2019).

Research on Qigong mechanisms of action models Eastern exercise toward Western theory, including stimulation of meridians through mental intent, acupressure, and self-massage [9]. This article will focus on the breathing-adjustment of Qigong for the adjustment of body state, which offers an integrating point to a broad spectrum of establishing artificial intelligence (AI)-Medicine training databases, thereby providing a vitality score system for breathing patterns with different pathologies unique to individuals.

## 2. Clinical Significance

Breathing-adjustment is an essential component of Qigong. It includes external breathing, the exchange of external inspired and expired air interfacing with blood flow in the lungs, and internal respiration represented by the exchange of gasses between blood and peripheral tissues. The primary purpose of Qigong breathing is to adjust external breathing for better lung function. Potential utilization of respiratory function in normal breathing represents less than 20% capacity of lung function. The unused lung potential may degenerate and even physically atrophy. Dysfunctional lungs lead to a hypoxic microenvironment within tissues, thereby triggering hypoxia-induced cell injury or death in cerebral ischemia. This is the second leading cause of death and disability worldwide, most likely through mitochondrial-stress and caspase-dependent pathways [10], such as simultaneous downregulation of HIF-1α/FASN [11]. Lung diseases with hypoxia are complicated by pulmonary hypertension, leading to heart failure and death, the leading cause of death worldwide [12]. Other incurable chronic diseases of persistent inflammatory states are associated with hypoxic-microenvironment, resulting in loss of their adaptive responses and, eventually, the development of fibrosis and fixed microvascular deficits [13].

The deep breathing exercises of Qigong can revive the unutilized respiratory function of the lungs. Qigong breathing practices not only mediate a reduction in the inflammatory but also enhance anti-inflammatory gene expression [14]. Such Qigong-style profound breathing effects upon pulmonary function exhibited statistically improved body mass index (BMI) and vital capacity (VC), as shown by the time-resolved quantitative analysis of diaphragm motion with dynamic chest radiography in a cohort of 174 patients [15]. Qigong breathing exercises offer promising efficacy in patients with type 2 diabetes mellitus (T2DM), as shown in randomized, controlled pilot studies. These exercises also positively affect clinical parameters of type II Diabetes Mellitus, including blood glucose, triglycerides, total cholesterol, weight, BMI, and insulin resistance [16].

Of note: Qigong breathing differs from Diaphragmatic Breathing. The diaphragm is a large, dome-shaped muscle located at the base of the lungs. A medical device to monitor diaphragmatic breathing help patients use the diaphragm correctly to cure asthma [17], from which more than six million children in the United States suffer. Qigong breathing can help medically underserved oncology patients [18]. Even though reports show the efficacy of Qigong practice for some patients; however, inconsistency has been noted because no standard procedure has been made. Some questions remain to be elucidated, such as “What can clinicians do to adopt this approach?”, “How is Qigong-dosage calculated?”, and “Can clients do Qigong on their own?”. All of these questions demand to study how Qigong regulates human health. The mechanism of action for the therapeutic breathing of the Qigong practice is not fully illustrated; however, we can have some glimpse into the functional aspects of Qigong, as follows.

## 3. Mechanism of Action for Practice of Breathing

Life can be defined as the constant process of absorbing and consuming energy, inhaling fresh air and nutrients, and exhaling spent gases and waste products. Specifically, breathing in enough oxygen (O_2_) is required for biological activity and survival of animals and humans. Lack of deprivation of O_2_ in local tissues or the whole-body (termed hypoxia) causes the metabolic crisis, affecting physiological functions and viability [19]. The main goal of the Qigong breathing-adjustment is to exercise the lungs to improve the efficiency of the absorptive potential of the respiratory system. Anions and activated O_2_ in the air are known as “air vitamins,” which have the functions of cleaning blood, increasing cell activity, and enhancing immune function [20]. During Qigong breathing exercises, fingers may feel distended, which is believed to represent improved O_2_ delivery throughout the microcirculation of the human body [21].

Modern medicine has proved that adequate oxygen can delay the aging process and disease onset of the heart, brain, lung, spleen, liver, kidney, and other essential organs of the human body. Qigong exercises can be preventing or even curing disease and reduce the chance of sudden onset of persistent diseases. Breathing-adjustment requires exercise in a peaceful state. It requires the practitioner to coordinate body–mind–spirit balance to keep the body relaxed in a natural state. It requires the practitioner to breathe gently, reducing oxygen consumption and decreasing heart rate and blood pressure. Qigong practitioners practice breathing exercises affecting different functional states, including running-Qi, nourishing-Qi, and emitting-Qi.

The training of mind adjustment, breathing adjustment, and body adjustment coordinate mind-body-shape for the human body through the exercise and then make the body more energetic and coordinate well. On the other hand, through the coordination exercise between mind and body, Qigong training can improve the function of tissues and organs and enhance physical fitness. Through the balanced movement of the mind and body, the human body is prevented from deteriorating, and the functions of the tissues and organs of the body are improved from the inside radiating outward. In the end, it will achieve the purpose of enhancing physical fitness, preventing disease and curing diseases, improving human immunity, and promoting physical and mental health. Through long-term adherence to Qigong Breathing practice, humans can improve and alleviate diseases such as cardiovascular and cerebrovascular conditions, respiratory system, cardiopulmonary function, and sub-health population.

Qigong is a traditional method for healthcare and disease preservation in China. The theory of meridians, acupoints, and Qixue (O_2_-blood flow) is the theoretical basis of Qigong in China. It is an integral part of traditional Chinese medicine (TCM). Indeed, retrospectively speaking, Confucianism, Taoism, Buddhism, and Wushu (Kungfu): All of these TCM theoretical schools have their points of view in the understanding of Qigong. The relationship between Qigong and Chinese traditional Wushu (Kungfu) is inseparable. Among the Chinese Wushu (i.e., Kungfu as known in Western culture), Hard Qigong (a type of Kungfu to strengthen the muscle elasticity and toughness) shows some of the most amazing results. With the development of science and technology, different knowledge and many kinds of techniques can be used to investigate and understand the mechanisms of action of Qigong and to explore more effects of Qigong for a better benefit to the people.

Interestingly, to determine the mechanism of Qigong’s action in physics, a physical form of Qigong breathing is captured as a ‘Ki-beam’ that carries ‘entropy’ (or information). Such Ki-energy exhibits near-infrared radiation, the wavelength is between 800 and 2700 nm, with peak around 1000 nm, showing a ‘sensitive’ distance of 100 m,, as measured by the Stimulated Emission of Radiation’ (i.e., LASER) for the generation of Ki-energy, from 37 Qigong participants in the United States through the Nishino Breathing Method [22]. Qigong breathing can lead the natural flow of Ki through the body to achieve harmony for the effects of complementary and alternative medicine [23]. Qigong breathing allows a practitioner to emit Ki-energy in the NIR spectrum, which can travel 100-m and mediate physical effects upon targets within that diameter.

Along the line of above normal physiology, a clinical trial (NCT02060123), which enrolled 240 abused women, aged ≥18 years, who suffered from chronic psychological stress, showed shorter telomeres than non-abused women. Amazingly, upon Qigong exercise, the abused women obtained the increased activity of telomerase, an enzyme that counters telomere shortening [24]. The findings indicate that Qigong breathing practice behaviors can relieve the effects of intimate partner violence (IPV)-related psychological stress on health, and slow down cellular aging via telomerase.

Another report presented results on sixty-four participants that were randomly assigned to either a Qigong breathing group or a control group, measuring fatigue symptoms, physical functioning, mental functioning, and telomerase activity. They found that “Fatigue symptoms and mental functioning were significantly improved in the qigong group compared to controls. Telomerase activity increased in the qigong group from 0.102 to 0.178 arbitrary units (*p*  <  0.05). The change was statistically significant when compared to the control group (*p*  <  0.05).” Thus, they concluded, “Qigong exercise may be used as an alternative and complementary therapy or rehabilitative program for chronic fatigue and chronic fatigue syndrome” [25].

The other functional impacts of Qigong breathing include:

(1) Promoting active energy flow through the meridians of the entire body (heat generation to warm up the body for thermal therapeutics).

(2) Enhancing blood flow to oxygenate body organs for detoxication, such as removal of inflammatory molecules out of injured tissue loci.

(3) Influencing the tissue microenvironment through mechanosensing subcellular organelle like caveolae [26]—“Cells and their extracellular matrix exhibit a dynamic reciprocity in the growth and formation of tissue through mechanotransduction and continuously adapt to physical cues in their environment through gene, protein, and cytokine expression” [27].

(4) Emerging data showed that Qi is affected by certain pharmaceuticals, such as an SMS recipe that was comprised of Ginseng Radix (GR, a Qi-invigorating herb), Ophiopogonis Radix (OR, a Yin-nourishing herb), and Schisandrae Fructus (SF, an “astringent” herb). SF also possesses antioxidant and anti-inflammatory actions, which are likely to synergize the actions of GR and OR. The elucidation of the biochemical basis underlying the pharmacological actions of SMS (ShengMai San and its component herbs) can serve as a paradigm for the modernization of traditional Chinese herbal formulations used in treating disease [28]. What have we learned from acupuncture research for fibromyalgia (FM)? FM affects 1.75% of the population, which is characterized by widespread chronic pain in muscles and joints and associated with central sensitization of controlling irritable bowel, irritable bladder, and headache, fatigue, poor cognition, unrefreshed sleep, and mood disturbance. New evidence shows efficacy of Qi-treatment on all of these syndromes [18].

(5) Improving sleep is to help regeneration of tissue functions [29].

(6) Determining “cut-off values for neonatal SpO2 (reference values for oxygen saturation) at mild and moderate altitudes and to provide new values for pediatricians to refer to when screening neonates for severe congenital heart or lung diseases” [30].

All of these can allow deciding a better healthcare strategy.

## 4. Comprehensive Healthcare by Integrating Western Medicine with TCM

Western medicine and TCM often allude to one other, yet they have so far failed to directly converge, allowing for synergy of each other’s knowledge in advancing integrated theories. Strengthening this interaction benefits both disciplines: Western medicine constrains our understanding of the disease-focused approach, TCM can expand the Western view on medicine to enhance overall mind-body-spirit health to support healing and improve quality of life. Artificial Intelligence may provide a novel theoretical framework to accelerate the integration of TCM and Western clinical medicine theory and physiology.

Holistic care (TCM) supporting digital, artificial intelligence medicine (AI Medicine) may provide a novel tool for evaluating the efficacy and guiding the practices of Qigong, supplementing comprehensive human health. Like the practice of Yoga and Zen, Qigong breathing promotes the development of a healthier lifestyle. Key to leveraging an improved therapeutic appreciation of the currently marginalized perception of Qigong beneficial effects of Qigong breathing on health and diseases augmented by developing the concepts of “breathing signatures”, which defines our life breathing patterns, may promote a natural, pure and healthier breathing style for daily life. Specifically, the most visible AI-Medicine integrated algorithm will bridge both present realities of heart-beat and pulse measurement (related to breathing) and more speculative scenarios of breathing patterns for predictive and preventive medicine, leading to measure breathing signature and its derived vitality score index (VSI).

## 5. The Concept

**Figure 1:**
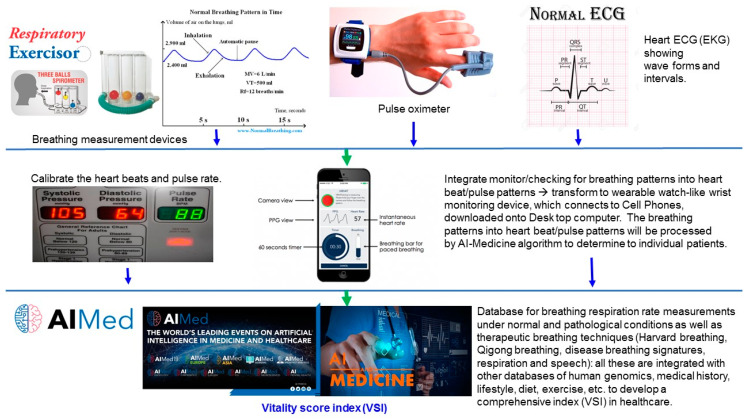
Development of a vitality score index (VSI), which is the collective strength of more-specialized signature breathing patterns unique for individuals. The VSI is derived from an AI Medicine algorithm that integrates physiology, heartbeat, pulse oximeter, blood pressure, wristwear, cell phone monitor, respiration and speech, lifestyle, diet, exercise, and breathing pattern. Such VSI data management not only assists our understanding of the underlying biological mechanisms of the whole body, but also predicts the outcomes of behaviors and medical treatment. A normal ECG records the electrical activity of the heart ((Electrocardiogram in English, or EKG—Elektro-kardiographie in German)): P wave (Atrial depolarization), PR segment, PR interval, QRS complex (QRS duration, Ventricular depolarization)), QT interval, ST segment, T wave (Ventricular repolarization), and U wave. Best Respiratory Spirometer Lung Exerciser Suppliers. Artificial intelligence in medicine (AI Medicine, AIMed) will change lives in many ways. Already, AI solutions are being deployed and having a significant impact on healthcare. (Credits: All images belong to Google images. AIMed logo designer: Anthony C. Chang, MD, MPH, MBA, at CHOC Children’s Hospital).

Monitoring a daily vitality score index (VSI) plays an indispensable role in disease prevention. The VSI can be defined by an algorithm that integrates all parameters, including heartbeat, pulse, breathing patterns, lifestyle, exercise, diet, and other physiological activities, derived from modern technology and AI-Medicine (below) (Figure 1). Currently, TCM practitioners can intuitively diagnose specific breathing signatures in health and disease. Physical breathing signatures of health and disease may prove discoverable through quantitative measurements as prescribed by Western clinical inquiry, like pulse oximeters and breath detection devices. The entire body and organ-specific surveillance of trends in data collections of an individual’s lifestyle and behavior, including dietary, and environmental exposures, disease treatment, genome and epigenome landscapes, all remain to be integrated with analysis of individual records for the pools of patients diagnosed with specific illness of pathological databases from many ethical population-based registries in many countries. As such a systematic analysis for an individual patient is processed with a daily operating algorithm, resulting in that a vitality score index (VSI) linked with breathing patterns as a therapeutic window [31] will surface and provide a subject inquired about predicting clinical outcomes of following specific breathing practice. Ultimately, AI-Medicine of integrating VSI will help to forecast life expectancy, years of life lost, and all-cause and cause-specific mortality. Thus, a simple task of breathing patterns, if recorded, aids a patient to maintain healthiness (Figure 1).

## 6. Technology and AI Medicine

Currently, the healthcare industry uses artificial intelligence (AI) to help turn breathing pattern data into knowledge-based medicine (AI Medicine), with the ultimate goal of using it to inform therapeutic interventions and improve patient outcomes (Table 1). Such a unique view of potentially connecting Eastern Medicine and Western Technology can add a novel insight into our current understanding of both Western and Eastern medicine (Figure 1).

A surge in the development of artificial intelligence (AI) technology is driving a new wave of open-source tools for analyzing human behavior, lifestyle, diet, and breathing patterns. Researchers have long been interested in tracking heartbeat, as heartbeat counting can be converted from the read-out of wrist pulse patterns (watch-like wrist belt). Conventionally, breathing patterns have involved spending hours recording breath-in and -out by desktop devices specific for inhale/exhale volume in a dedicated setting. Few tools can capture breathing patterns daily, like heartbeat/pulse in finer details. Each tool (heartbeat, pulse oximeter, breathing) has limitations; some require specific experimental set-ups or do not work well when there is a need to monitor daily. Nevertheless, advances in digital, image capture, and machine learning will improve AI-transformation alongside gathering of separated pieces of health information to translate breath-coordinates into behavior-based healthcare, essential for human life. AI/deep learning could integrate the comprehensive databases of a patient into a vitality score index (VSI), to help supervise their life.

How AI could develop a new understanding of how breathing patterns can diagnose diseases and how therapeutic breathing modalities (Qigong) could augment western medicine treatment strategies remain to be elucidated in order to define the VSI ranges for threshold values. Literature inspires specific directions, as illustrated from other fields. For example, an AI-Medicine tool (a speech-based algorithm, Random forest algorithm) has been used to objectively differentiate the diagnosis of PTSD ([42]. Similar to our proposed “breathing signatures” associated with diseases, a “voice signature” was sorted out of the diagnosis of 52 posttraumatic stress disorder (PTSD) cases from 77 controls, to create the PTSD-specific signature voice [42]. The “PTSD-specific signature voice” markers indicated slower, more monotonous speech, less change in tonality, and less activation than the controls (non-PTSD psychiatric disorders). Other speech signatures identified include the “pressured” voice sounds, which may indicate conditions such as bipolar disorder, while the voice characteristics of “monotone,” “lifeless,” and “metallic “speech may indicate depression.

Future studies should aim at the identification of breathing signatures associated with diseases. Therefore, speech-based techniques offer an alternative to conventional behavioral [44] and biological (cellular and molecular) biomarkers for PTSD, including alterations in neural structures and circuit functioning, cancer genome [45], genomics [46], neurochemistry, immune functioning, and psychophysiology [47]. Behavioral and biological (cellular and molecular) biomarkers exhibit inherited drawbacks in accuracy, cost, and patient burden, which preclude routine use in clinical practice. In practical, the dynamic change of a “voice signature” can be recorded in an automated diagnostic system, even used as a smartphone app, allowing several continuous, cheap, remote, and non-intrusive data collections. Such a voice database can be used to interpret health and disease conditions. Similar tools and devices can be established to record the dynamic patterning of Qigong breathing signatures, which can be elaborated with the following six examples:

First, to reflect on our intention to borrow the concept of “18 voice features” [42]—voice signature as the set up to rally out our speculation on “breathing signature.” We suggest that the speech recognition algorithm may be further developed into a breathing classifier, as speech and breath are related physically and physiologically, manifested in speech notes by Winston Churchill (the book, pp. 32–33, [48]). We speculate that the AI Medicine algorithm can integrate both voice speech and breath to another level of quantitively measurable parameters along the line of Qigong breathing techniques—in fact, Qigong practitioners change their voice features as a natural result of breathing signature changes.

Indeed, a previous study reported that “Using a spectral subtraction algorithm to remove scanner gradient noise from recorded speech, we related the timing of speech, stimulus presentation, chest wall movement, and image acquisition. We explored the relationship of an extended speech event time course and respiration on signal variance by performing a series of voxelwise regression analyses. Our results demonstrate that these effects are spatially heterogeneous, but their anatomic locations converge across subjects. Affected locations included basal areas (orbitofrontal, mesial temporal, brainstem), areas adjacent to CSF spaces, and lateral frontal areas. If left unmodeled, speech-related variance can result in regional detection bias that affects some areas critically implicated in language function. The results establish the feasibility of detecting and mitigating speech-related variance in rapid event-related fMRI experiments with single-word utterances. They further demonstrate the utility of precise timing information about speech and respiration for this purpose” [49]. These indicate that AI Medicine may further integrate speech and breath patterns to create a VSI score to monitor the health condition.

Another report came from “using speech analysis and machine learning techniques for obstructive sleep apnea (OSA) detection” for diagnostic applications [50]. “A large speech database including 426 male Spanish speakers suspected to suffer OSA and derived to a sleep disorders unit was used to study the clinical validity of several proposals using machine learning techniques to predict the apnea-hypopnea index (AHI) or classify individuals according to their OSA severity.” This data indicate that a pathological signature of breathing can be determined.

Third, as “The tongue is a critical organ for a variety of functions, including swallowing, respiration, and speech,” researchers used Diffusion tensor imaging (DTI) to reconstruct tongue muscle fiber tracts, and concomitantly applied “high angular resolution diffusion imaging (HARDI) and diffusion spectrum imaging (DSI) to reconstruct the crossing fibers that occur where the tongue muscles interdigitate, which is a large percentage of the tongue volume [41].” They further computed with anatomical knowledge of tongue muscles toward fiber directions as “estimated within a maximum a posteriori (MAP) framework, and the resulting objective function is solved using a noise-aware weighted ℓ1-norm minimization algorithm,” thereby creating the potential clinical use for this imaging and image analysis methodology. Thus, in-depth analyses can reach a conclusion how tongue physiology impacts breathing patterns.

Fourth, research shows that “frequency of swallowing may serve as a predictor for detecting food intake, differentiating liquids and solids, and estimating ingested mass [51]”. Their results suggest “high efficiency of the proposed methodology in the separation of swallowing sounds from artifacts that originate from respiration, intrinsic speech, head movements, food ingestion, and ambient noise.” Only AI Medicine can help to collect big data sets to sort out for these patterns.

Fifth, audiovisual emotion manifested in facial expression or speech got tied in with physiological signals for emotion recognition, which can be tracked down by current technologies. Such technologies made into a report [52]: “Four-channel biosensors were used to measure electromyogram, electrocardiogram, skin conductivity, and respiration changes. A wide range of physiological features from various analysis domains, including time/frequency, entropy, geometric analysis, subband spectra, and multiscale entropy, is proposed in order to find the best emotion-relevant features and to correlate them with emotional states. The best features extracted are specified in detail, and their effectiveness is proven by classification results. Classification of four musical emotions (positive/high arousal, negative/high arousal, negative/low arousal, positive/low arousal) is performed by using extended linear discriminant analysis (pLDA). Furthermore, by exploiting a dichotomic property of the 2D emotion model, we develop a novel scheme of emotion-specific multilevel dichotomous classification (EMDC) and compare its performance with direct multiclass classification using the pLDA. The improved recognition accuracy of 95% and 70% for subject-dependent and subject-independent classification, respectively, is achieved by using the EMDC scheme.” All of these technologies can be integrated for comprehensive reports of wellbeing in an individual.

Sixth, as larynx musculature compromises breathing and speech function, researchers design algorithms for differential thresholds of the physiopathology of larynx musculature to record spectra unique for either breathing or speech function by using electric stimulation and electromyography for muscle contraction in pattern recognition [53]. This larynx musculature changes can be linked to Qigong breath recording.

AI does broadly around potential social and economic impacts even though most people do not know what microservices architecture is, for example, even if some of the applications (Pulse Oximeter, Heart-beating meter, Watson—IBM Google machine/quantum computers [54]) (Table 1) they use every day were built in decoupled fashion, yet to be integrated into AI Medicine platforms (Figure 1), such as Algorithm to predict risk of readmission [55] and AI algorithm to predict sepsis in a cohort of 204,485 infants [56]. National Academies of Sciences, Engineering, and Medicine consensus committee report “defining 5 priority development areas for the US health care system to integrate social care into health care delivery: Designing delivery systems and building workforce, developing digital infrastructure interoperable between health and social care organizations, and funding the integration and research into the effectiveness and implementation of social care practices in health care settings. “In the contemporary AI field, deep learning implies a deep neural network, which allows intertwining among these factors.” Indeed, these assessments can be integrated and translated into the determination of VSI scores.

## 7. Conclusions

We hypothesize that Qigong breathing, if in conjunction with daily practicing, will improve outcomes in patients with diverse pathologies. As a test of this concept, we propose to implement a cross-institutional prospective study that uses Qigong breathing exercise as an integrated point of health assessment and appropriate combination therapies to associate the “breathing signature” with immune system biomarkers [57], thereby defining a vitality score index (VSI), an indication that can be daily monitored. Such an association study would establish a tracking pattern [58] to allow the definition of a “therapeutic window” [31] for a spatiotemporal assessment [59] to direct long-term care strategy. However, the breakthrough will come from establishing an AI-Medicine training camp of databases, which will integrate the connection of Qigong-like breathing patterns with individual pathology. Such an integrated connection by AI Medicine algorithms will identify breathing patterns and guide the medical intervention. All these eventually lead to support the state of health for personalized care by gravitating toward the development of a vitality score index (VSI), which is the collective strength of more-specialized signature breathing patterns unique for individuals. The VSI will be derived from an AI Medicine algorithm that integrates the physiology, heartbeat, pulse oximeter, blood pressure, wristwear, cell phone monitor, lifestyle, diet, exercise, and breathing patterning (Figure 1). Such VSI data management will provide not only our understanding of the underlying biological mechanisms of the whole body but also predicts the outcomes of behaviors and medical treatment.

## Figures and Tables

**Table 1:** Artificial intelligence-integrated health management of breathing patterns and heartbeat/pulse patterns.

Technology of Medical Measurement	Evidence-Based Wellness and Maintenance	Disease-Centric Parameters of Personalized Strategies	References
Hypoxia	hypoxia-inducible factors (HIFs).	hypoxia-ischemia	[19]
Local/regional hypoxia	Hippocampus	CA3 pyramidal neurons	[6]
Whole-body hypoxia	Heart functions	myocardial infarction	[4]
Breathing patterns	Non-Invasive Stretchable and Wearable Respiratory Rate Sensor for respiration rate		[32]
	e-Health nasal sensor (consists of a passive and non-invasive single-lead electrocardiogram (ECG) acquisition module and an ECG-derived respiratory (EDR) algorithm in the working prototype of a mobile application)		[33]
		Nose breathing vs. mouth breathing (correlations between mouth breathing and cognition show that decreased oxygen saturation during mouth breathing results not only in morphological deformations but also in poor learning outcomes)	[34]
Heartbeat/pulse patterns		Flattening of the flow velocity (pulse) patterns correlates with the local severity of arteriosclerotic disease	[35]
	Preventive medicine using pulse oximetry screening		[36]
		Pulse transit time (PTT) is the time it takes a pulse wave to travel between two arterial sites (R-wave-gated photo-plethysmography (RWPP) as of measurement of PTT as a surrogate for intra-thoracic pressure changes in obstructive sleep apnea)	[37]
		Pulse Oximetry Screening for Critical Congenital Heart Defects	[38]
AI-Medicine algorithm			
	Algorithm to track changes in cardiorespiratory interactions (heartbeat intervals and respiratory recordings under dynamic breathing patterns)		[39]
		Respiratory sinus arrhythmia (RSA) with algorithm for quantifying instantaneous RSA as applied to heartbeat interval and respiratory recordings in order to track changes in cardiorespiratory interactions elicited during meditation, otherwise not evidenced in control resting states)	[40]
	Tongue is a critical organ for respiration and speech		[41]
		18 voice features with posttraumatic stress disorder	[42]
	Breathing pattern parameters: Peak airway pressure (Paw_peek_), mean airway pressure (Paw_mean_), tidal volume (V_T_, mL/kg), minute volume (MV), respiratory muscle unloading (peak electricity of diaphragm (EAdi_peak_), P 0.1, V_T_/EAdi), clinical outcomes (ICU mortality, duration of ventilation days, ICU stay time, hospital stay time		[43]
